# Age- and Wavelength-Dependency of *Drosophila* Larval Phototaxis and Behavioral Responses to Natural Lighting Conditions

**DOI:** 10.3389/fnbeh.2017.00066

**Published:** 2017-04-20

**Authors:** Tim-Henning Humberg, Simon G. Sprecher

**Affiliations:** Department of Biology, University of FribourgFribourg, Switzerland

**Keywords:** visual system, phototaxis, navigation, *Drosophila* larva, photoreceptor

## Abstract

Animals use various environmental cues as key determinant for their behavioral decisions. Visual systems are hereby responsible to translate light-dependent stimuli into neuronal encoded information. Even though the larval eyes of the fruit fly *Drosophila melanogaster* are comparably simple, they comprise two types of photoreceptor neurons (PRs), defined by different Rhodopsin genes expressed. Recent findings support that for light avoidance Rhodopsin5 (Rh5) expressing photoreceptors are crucial, while Rhodopsin6 (Rh6) expressing photoreceptors are dispensable under laboratory conditions. However, it remains debated how animals change light preference during larval live. We show that larval negative phototaxis is age-independent as it persists in larvae from foraging to wandering developmental stages. Moreover, if spectrally different Rhodopsins are employed for the detection of different wavelength of light remains unexplored. We found that negative phototaxis can be elicit by light with wavelengths ranging from ultraviolet (UV) to green. This behavior is uniquely mediated by Rh5 expressing photoreceptors, and therefore suggest that this photoreceptor-type is able to perceive UV up to green light. In contrast to laboratory our field experiments revealed that *Drosophila* larvae uses both types of photoreceptors under natural lighting conditions. All our results, demonstrate that *Drosophila* larval eyes mediate avoidance of light stimuli with a wide, ecological relevant range of quantity (intensities) and quality (wavelengths). Thus, the two photoreceptor-types appear more likely to play a role in different aspects of phototaxis under natural lighting conditions, rather than color discrimination.

## Introduction

The ability of an animal to navigate in response to distinct environmental cues depends on proper perception and processing of sensory stimuli. Light is perceived by specialized photoreceptor neurons (PRs) in the eye, which transmit this information to defined neural circuits in the brain. In many animal species light is perceived as a highly attractive or aversive cue, depending on their life style. The larva of the fruit fly *Drosophila melanogaster* spends most of its life immersed feeding on rotting fruits. While larvae are attracted towards olfactory stimuli, they are strongly repelled by light and seek for dark or less light-exposed surroundings. While this negative phototaxis behavior has been well documented, it remains less clear how the animal adapts its light preference during its larval life stage. The larval eye is composed of two PR-subtypes either expressing the blue-tuned Rhodopsin5 (Rh5) or the green-tuned Rhodopsin6 (Rh6) receptor gene (Salcedo et al., 1999; Malpel et al., 2002; Sprecher et al., 2007; Sprecher and Desplan, 2008). For phototaxis only Rh5-PRs are essential, while no direct role for Rh6-PRs has been identified in rapid light-avoidance (Hassan et al., 2005; Keene et al., 2011; Kane et al., 2013). This raises the question how larvae react to different wavelengths of light and what the function of Rh6-PRs for visually guided behaviors is.

In the current study, we investigate age- and wavelength-dependency of visual navigation and identify a role of Rh6-PRs for phototaxis in natural lighting conditions. First studies on larval phototaxis were performed over a century ago and several different hypotheses on age-dependency have been supported in the past years. More than 35 years ago it was reported that *Drosophila melanogaster* larvae are photophilic in early stages, photoneutral in later stages, and photonegative in the end of the larval life (Manning and Markow, 1981; Markow, 1981). In contrast to this early description, a general consensus nowadays in most of the reports is that *Drosophila* larvae are photophobic during early stages of development (Godoy-Herrera et al., 1992; Sawin-McCormack et al., 1995; Busto et al., 1999; Warrick et al., 1999; Hassan et al., 2000; Mazzoni et al., 2005; Scantlebury et al., 2007; Gong, 2009; Keene et al., 2011; von Essen et al., 2011; Yamanaka et al., 2013). However, how phototaxis is altered or maintained during later stages remains debated. One scenario is that larvae become photoneutral (Sawin-McCormack et al., 1995) or even photophilic with age (Godoy-Herrera et al., 1992). Alternatively animals may remain photophobic throughout the larval development including wandering third instar stages (Yamanaka et al., 2013). Interestingly, despite strong experimental evidence the notion that larvae switch from photonegative to photoneutral appears predominant, presumably since at late larval stages larvae leave the “dark” food and move towards “light” for pupation. Most studies on larval photobehavior were based on light/dark choice assays, in which the experimenter is counting the number of larvae in the dark and in the illuminated region after a certain time. Such behavioral assays do not provide detailed information on the aspects of larval behavior, which are changed in response to light-exposure. Recently using high-resolution computer-based tracking analysis, the navigational logic of visual navigation from sensory input to motor output was investigated (Kane et al., 2013). Larval navigation is based on a combination of forward movements (called runs) and reorientations (called turns) (Luo et al., 2010; Gomez-Marin et al., 2011; Lahiri et al., 2011; Gershow et al., 2012; Kane et al., 2013). Changes in light-intensity elicit turning behavior (Busto et al., 1999; Hassan et al., 2000, 2005; Scantlebury et al., 2007; Kane et al., 2013). During these turns larvae move their head from one side to the other (called head-sweep) to probe the environment (Busto et al., 1999; Hassan et al., 2000, 2005; Scantlebury et al., 2007; Kane et al., 2013). Thus information about the light intensity is integrated temporally and not spatially (Kane et al., 2013). A rejected head-sweep is followed by another head-sweep and an accepted head-sweep is followed by a run in this new direction (Kane et al., 2013). It has been demonstrated, that for efficient phototaxis, larvae bias the turn direction away from the light source and make bigger turns while heading towards the light source (Kane et al., 2013). Using a computer-based tracking system, we found that *Drosophila* larvae are photonegative and robustly navigate away from a light source throughout all tested larval developmental stages. Moreover, we did not observe any influence of larval age on distinct phototaxis navigation parameters. These results further corroborate a model in which visual cues transmitted by the larval eye result in avoidance behavior throughout larval life.

While Rh5 is tuned towards blue and Rh6 towards green, the calculated absorption spectra of the two Rhodopsins are overlapping and also include absorption peaks at different wavelengths (Salcedo et al., 1999). Even though photonegativity of* Drosophila* larvae can be elicited by light with wavelengths ranging from ultraviolet (UV) to green (Warrick et al., 1999), white light avoidance is purely mediated by Rh5-PRs, whereas Rh6-PRs are dispensable for this behavior (Hassan et al., 2005; Keene et al., 2011; Kane et al., 2013). If Rh6-PRs are necessary for phototaxis under green light or even under other conditions, remained unclear. By using different nearly monochromatic illumination sources, we found that *Drosophila* larvae navigate away from a wide range of wavelengths (UV-A to green). Moreover, we observed that the negative phototaxis in response to these monochromatic light sources is mediated by the larval eye or more precisely solely by Rh5-PRs. Our data suggests that larval Rh5-PRs wavelength sensitivity is much wider than only “blue” light. Moreover, our outdoor experiments revealed a functional role of Rh6-PRs in light avoidance. Surprisingly, under sunny conditions both PR-subtypes alone were sufficient to mediate light avoidance, whereas under cloudy conditions both PR-subtypes were necessary for successful light avoidance. Our experiments demonstrate that the larval eye is necessary for mediating light avoidance over a wide, biologically relevant range of light intensities. Despite expression of spectrally distinct Rhodopsins, the two larval PR-subtypes appear not to function in color-dependent behaviors but likely rather contribute to distinct aspects in the navigational behaviors of the larva.

## Materials and Methods

### Fly Strains

Behavioral experiments were performed with larvae of the following lines: wild-type (WT) *Canton-S* (courtesy of R. Stocker), *w*^−^ ; Rh5^2^, w^−^ ; Rh6^1^, w^−^ ; Rh5^2^ ; Rh6^1^ (courtesy of C. Desplan) and *w*^1118^; Mi((ET1))Gr28b^MB03888^ (Bloomington stock number 24190).

*Drosophila melanogaster* flies and larvae were raised on cornmeal medium supplemented with molasses, fructose and yeast at 25°C in a 12 h light −12 h dark cycle. Adult flies were allowed to lay eggs for 4 h (for age-dependency experiments) or 24 h (for all other experiments) and then flipped to a fresh food vial. One day after egg laying (AEL) the first larvae start hatching. Age-dependency experiments were performed on foraging larvae (2, 3, 4 and 5 days AEL) as well as on wandering larvae (6 days AEL). All the other experiments were performed on foraging third-instar larvae (4 days AEL). Foraging larvae were collected out of the food and wandering larvae were collected from the walls of the vials.

### Preparation of Behavior Experiments

All experiments were performed on 2% agarose plates. 2% agarose (Agarose Standard, Roth) was filled into 24.5 × 24.5 cm plates (BD Falcon BioDishXL, BD Biosciences). At the bottom of these plates we placed a black aluminum plate, in order to increase the contrast. The agarose had to cool down to room temperature before experiments were performed.

At least 20 min before the experiment started food vials (containing larvae) were stored in darkness. With a spoon larvae were transferred into a petri dish. With tap water the larvae were cleaned from the food. With a fine paintbrush 30 larvae were collected and kept in a water drop for up to 10 min. The 30 larvae were transferred into the middle of the agarose plate. All experiments were prepared under red illumination. We performed 10 trials per genotype and assay. The only exception were the outdoor experiments, in which we were comparing light avoidance in the morning (3 trials) and in the afternoon (4 trails).

### Tracking System

We used a computer-based tracking-system comparable to one described earlier (Gershow et al., 2012; Kane et al., 2013). The experiments were performed in a dark box, to prevent larvae from unwanted stimuli. For uniform illumination, the agarose plate was surrounded by red light emitting diodes (LEDs) (623 nm, Conrad). For 11 min larvae moved freely on the testing plate. The larval movements were recorded at 13 frames/s with a camera (acA2500–14 gm, Basler AG) equipped with a lens (Fujinon HF12.5HA-1B 12.5 mm/1.4, Fujifilm) and a red light bandpass filter (BP635, Midwest Optical Systems) from 45 cm above the center of the agarose plate.

For image acquisition we used a customized LabView software (Gershow et al., 2012; Kane et al., 2013). For analysis of larval behavior, we used the customized MAGATAnalyzer (Gershow et al., 2012; Kane et al., 2013). Further analysis and visualization of the data were performed with GraphPad, MatLab and R. During the first minute of each experiment larvae were allowed to acclimatize to the testing conditions and therefore this minute was not taken into consideration for analysis.

### Visual Stimulation

In the tracking system, we used for visual stimulation of the larvae either a projector (EX7200 Multimedia Projector, EPSON) equipped with a bandpass filter (335–610 nm, BG40, Thorlabs) or different LEDs emitting nearly monochromatic light. The projector produced light with an intensity of 2687 μW/cm^2^. Maximum intensity peaks were at 438 nm with half-widths of 12 nm (blue) and at 549 nm with half-widths of 12 nm (green). The projector was 38 cm away from the middle of the testing plate in a height of 26 cm and orientated with an angle of 40° with respect to the plate.

The colors, intensity peaks and half-widths of the LEDs were: UV-A (368 nm, half-widths: 7 nm), blue (466 nm, half-widths: 11 nm), green (514 nm, half-widths: 17 nm), yellow (595 nm, half-widths: 7 nm) and white (441 nm, half-widths: 13 nm; 586 nm, half-widths: 62 nm). The LEDs were placed 14 cm away from the middle of the agarose plate in a height of 10 cm over its surface and were orientated with an angle of 40° with respect to the plate. The different types of LEDs emitted light with an intensity of 72 μW/cm^2^ (UV-A), 71 μW/cm^2^ (blue, yellow and white) and 69 μW/cm^2^ (green). The “no stimulus groups” are WT larvae (4 days AEL, if not indicated differently), which were not stimulated with a directional light source.

### Phototaxis Navigation Strategies

From the recordings generated in the tracking system, we extracted and determined larval position, bearing, body contour, center of mass, position of head and tail and midline of the larvae as previously described (Scantlebury et al., 2007; Kane et al., 2013). Briefly, periods of forward locomotion, in which the larval head and body were aligned, were defined as runs. Time frames of slow or no forward movement were defined as turns, when they were coupled with head-sweeping behavior, or defined as pauses, when the head remained aligned with the body. The threshold speed, which defines stopping or starting a run, is calculated individually for each larva. These calculations are based on data points of time frames right before and after turn events. In case larval speed is slower than the mean speed right before and after turns, than this event is flagged as pausing (head is aligned with the body) or turning (head is not aligned with the body).

We used a compass for describing the heading direction of larvae. 0° indicates heading towards the light source and 180° indicates heading away from the light source. ±90° indicates heading perpendicular to the light source. All four directions were binned in 90°. Like this heading between −45° and +45° was defined as heading towards the light source (0°, +x direction), whereas heading between +135° and −135° was defined as heading away from the light source (180°, −x direction). Heading between +45° and +135° was defined as heading perpendicular to the light source (+90°, +y direction). And heading between −135° and −45° was defined as heading perpendicular to the light source (−90°, −y direction). We calculated a navigation index, in order to analyze the general phototaxis navigational performance. Therefore, the velocity of all larvae in x-direction was divided by the mean run speed in all directions. The navigation index would be −1, if all larvae would navigate uniformly away from the light source. If all larvae would run uniformly towards the light source, the navigation index would be +1. In case the run direction would be unbiased away and towards the light source the navigation index would by 0.

Furthermore, we analyzed the turn direction of larvae which were previously running perpendicular to the light source. If the heading direction before the turn was +90° a turn towards the right was considered as turning towards the light source, whereas a turn to the left was contemplated as a turn away from the light source, and vice versa for previous heading direction to −90°. In these cases a turn to the right was considered as a turn away from the light source, whereas a turn to the left was contemplated as turn towards the light source.

To analyze the navigational parameter “turn magnitude”, we compared the turn size of larvae, which were running previously towards (0°) or away from the light source (180°). The difference between heading before and after the turn was defined as the turn size.

To reveal defects in forward locomotion, we draw manually a virtual circle of 5 cm in diameter around each larva of the 5 and 6 days AEL data sets. The center of the circle was positioned on the respective larva. Larvae which left the circle with the whole body at least once throughout the complete 11 min of the experiment were counted as larvae with normal locomotion whereas larvae which failed to leave the circle were counted as larvae with decreased locomotion.

### Outdoor Experiments

All outdoor experiments were performed 636 m above the sea level at latitude N 46°47′34.64″ 46.79296° and longitude E 7°9′21.55″ 7.15599°. The experiments were performed between sun altitudes of 19° and 56°. For each experiment 30 larvae were prepared and placed on a 2% agarose plate like described above. The agarose plate was orientated with two borders perpendicular to the sun. Experiments were performed on either clear sunny days or when a layer of clouds were covering the sun. We divided the plate in a neutral zone and a solar and an antisolar zone. The neutral zone was the midline of the plate plus 1 cm in both directions and therefore also orientated perpendicular to the sun position. All larvae where placed at the beginning of the experiment in the neutral zone. Larvae which start in the neutral zone and were navigating away from the sun would end up in the antisolar zone. Larvae which were moving towards the sun would end up in the solar zone. At the end of the experiment we count how many larvae ended up in the different zones of the plate. During outdoor experiments larvae seemed to be less agile/slower than during indoor experiments. After 10 min larval light avoidance behavior was observable (for example for WT larvae), but larvae were still close to the starting region. To give to the larvae more time to navigate away from the starting point (in any direction), we increased experimental time to 20 min. For 20 min larvae could move freely on the plate. We did experiments during two distinct time windows in a day to minimize the effects of the environment. During our experiments the sun has an azimuth of 91°–140° in the morning and of 235°–261° in the afternoon. Between this two time windows the solar and antisolar side changed positions as the position of the sun changes during the day. For each experiment we calculated a preference index by subtracting the number of larvae in the antisolar side from the number of larvae in the solar side divided by the total number of larvae. The temperature of the testing plate was varying between the different experiments (cloudy conditions: 15–23°C and sunny conditions: 13–24°C) and sometimes also during single experiments by up to 4°C. However, the temperature was not varying at a given time point between different points of a testing plate. Experiments of WT and *Rh6* mutant larvae were always performed in parallel, as well as experiments of gustatory receptor 28b mutant larvae (*Gr28b*) and *Rh5*, *Rh6* double mutant larvae. Experiments of *Rh5* mutant larvae were performed in parallel with either one or the other of the two experimental sets.

### Tube Assays

We used glass tubes, which were 10 cm long and were 1.6 cm in diameter. Two glass tubes were fixed apposed together with transparent tape. We subdivided the two tubes in four sections (two dark areas and two enlighten areas) similar to the tube assay used by Sawin-McCormack et al. (1995). We used black tape to create the dark areas. All areas were 5 cm in length. Larvae were stimulated from above with the white LEDs. In one set of the experiments we placed the larvae at the beginning in the middle of one light area, comparable to the assay used by Sawin-McCormack et al. (1995), and in the other experimental set we placed the larvae as close as possible to the light-dark boundary comparable to the assay described by Yamanaka et al. (2013). In case of the last experimental set-up, five larvae were placed close to the light-dark boundary but still in the light area and five larvae were placed close to the boundary, but in the dark area. We performed 10 experiments each with 10 wandering larvae of age 6 days AEL for each experimental set-up. We calculated for each experiment the preference index after 5 min and after 10 min.

### Statistical Analysis

All data is presented as means and error bars indicate ± SEM. Statistical analyses were performed using standard statistic functions in MATLAB. An unpaired *t* test was used for testing the means per trial of navigation indices, preference indices, turn size bias, larval body size, mean run length, mean run speed, mean run speed/body size ratio, pause rate and turn rate of different groups against each other. Fisher’s exact test was used for testing the probability of turn direction against respective no-stimulus controls. A paired *t* test was used to test the mean preference indices after 5 and 10 min of the respective tube-assay set-ups against each other. A one sample *t* test was used to test the mean preference indices of the tube assays and the outdoor experiments against chance. Rejection of the null hypothesis that means were the same or that the mean is chance: *p < 0.05, **p < 0.01, ***p < 0.001. The Benjamini Hochberg procedure was used to adjust *p*-values in case of multiple comparisons.

## Results

### Larvae of all Developmental Stages Navigate Away from Light

**Figure 1:**
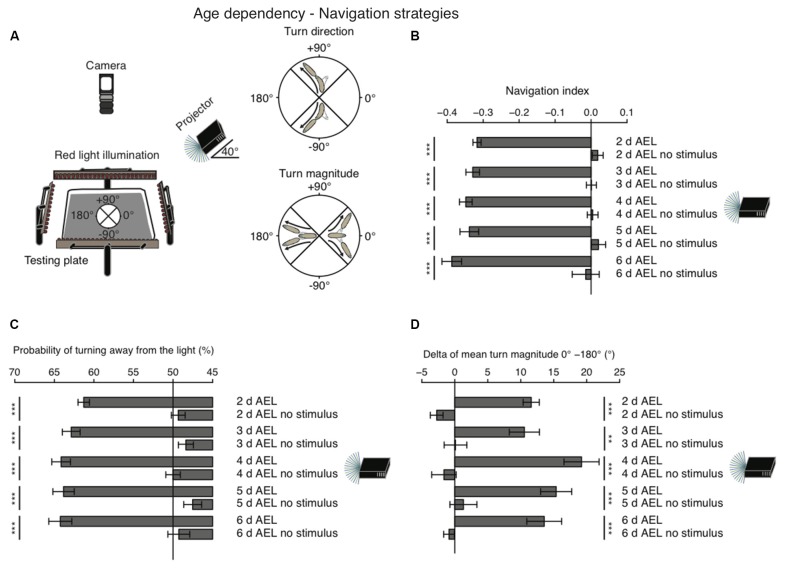
**Larval phototaxis is robust throughout all developmental stages. (A)** Schematic illustration of the behavioral set-up and larval phototaxis strategies. 30 larvae were placed in the center of the testing plate. The light source (in this case a projector) was positioned on one side of the testing plate. We used a navigational compass. Larvae heading towards the light source would head towards 0°. Heading away from the light source would be heading to 180° and a perpendicular orientation towards the light source would be towards +90° or −90°. Red light illumination was needed for the camera, which recorded larval behavior from top. Orientated perpendicular towards the light source, larvae are able to bias their turn direction away from the light source. Larvae make greater turns, when previously heading towards the light, compared with turns, when previously heading away from the light. **(B)** Navigation indices of foraging larvae (2, 3, 4 or 5 days after egg laying (AEL)) and wandering larvae (6 days AEL). Rejection of the null hypothesis that the data set and the no stimulus data set have the same mean: ***p < 0.001, unpaired *t* test. **(C)** Probability of turn direction after runs heading perpendicular to the light source. The Fisher’s exact test was performed to test that the probabilitiy of turning away from the light source is significantly different from turn direction probability of the respective no stimulus group: ***p < 0.001. **(D)** The difference of turn size of reorientations where runs were heading previously towards and away from the light source (turn size previously heading towards the light source—turn size previously heading away from the light source). Rejection of the null hypothesis that the data set of the stimulated animals and of the no stimulus group have the same mean: **p < 0.01, ***p < 0.001, unpaired *t* test.

When given a choice, *Drosophila* larvae prefer shaded over light-exposed areas. While it is generally agreed that larvae are initially repelled by light, it remains still debated, if during late larval stages photo-avoidance persists, if animals become photoneutral or if they even become photophilic (Godoy-Herrera et al., 1992; Sawin-McCormack et al., 1995; Yamanaka et al., 2013). In order to investigate visual navigation of larvae with different age, we used a previously developed computer-aided video tracking system (Gershow et al., 2012; Kane et al., 2013). Per experiment 30 larvae were placed on a testing plate and stimulated by a directional light source from one side (Figure 1A). A camera from top was recording larval behavior (Figure 1A).

As a first assessment of phototaxis we calculated a navigation index. The navigation index is defined as the average velocity along the *x*-axis (towards and away from the light source) divided by the average speed in total (see “Materials and Methods” Section). In such a fashion, larvae that move in a straight line away from the light source have a navigation index of −1, while animals that move directly in a straight line towards the light source have a navigation index of +1. Animals moving perpendicular to the light source or unbiased towards and away from the light source have a navigation index of 0. We found that during all stages larvae navigate robustly away from the light source, with negative navigation indices of −0.32 to −0.39 (Figure 1B). Surprisingly, larvae of the wandering stage (6 days AEL) showed the highest navigation index. However, the scores of different age groups are not significantly different (Figure 1B). In order to efficiently navigate in this assay larvae have been shown to use two navigation strategies (Kane et al., 2013). First, animals bias the turn magnitude: larvae make significantly larger turns when moving towards the light, while they make smaller turns when running away from the light source (Kane et al., 2013; Figure 1A). Second, animals bias the turn direction: during a reorientation event animals are more likely to initiate a run away from the light source than towards a light source (Kane et al., 2013; Figure 1A). Similarly, to the navigation index we did not observe significant differences of both behavioral parameters in different age groups. All tested groups were able to bias the probability of turning away from the light source and made bigger turns when heading towards the light as compared with turns performed while heading away from the light source (Figures 1C,D). Thus, these results support that there is no change of visually guided navigation towards late larval stages.

**Figure 2:**
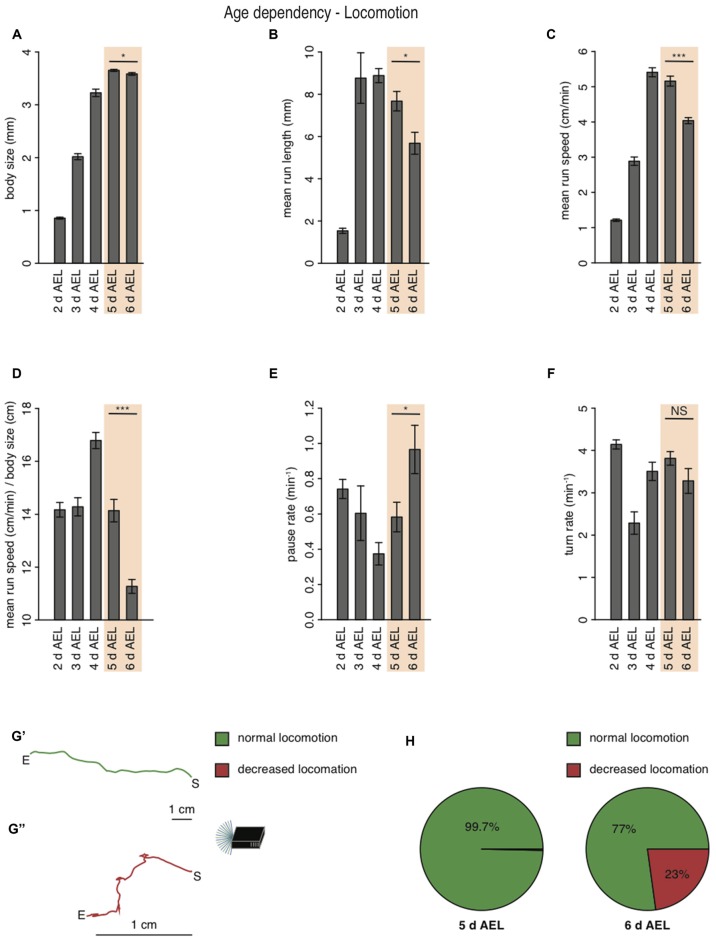
**Larval locomotion is changing with larval body size and age. (A)** Mean body size of different larval age groups. Mean run length **(B)**, mean run speed **(C)** and the calculated ratio of mean run speed to body size **(D)** of different larval age groups. Pause rate **(E)** and turn rate **(F)** per min of the different aged larval groups. **(A–F)** Data show the mean and error bars indicate ± SEM. Rejection of the null hypothesis that the data set of two groups have the same mean: *p < 0.05, ^***^p < 0.001, unpaired *t* test. **(G)** Two representative larval tracks of larvae corresponding to the age 6 days AEL. A larval track consists of all runs and turns the respective larvae is performing during the whole experiment. One track belongs to the category “normal locomotion” **(G′)**, whereas the other track belongs to the category “decreased locomotion” **(G″)**. “S” indicates the starting and “E” the end point of the respective larval track. Decreased locomotion was defined as loss of the ability to leave a virtual circle with 5 cm in diameter throughout the experiment. **(H)** Pie chart showing the percentage of larvae showing normal (green) and decreased (red) locomotion for larvae of age 5 and 6 days AEL.

During larval life, the animal drastically increases body length from 0.9 mm early to 3.2 mm at 4 days AEL (Figure 2A). During late developmental stages larval body length is greatest with 3.7 mm at 5 days AEL and decrease slightly again to 3.6 mm at 6 days AEL (Figure 2A). Interestingly, also the larval mean run length was reduced in wandering larvae compared to foraging larvae of age 5 days AEL (Figure 2B). With the increase of body length, we also observed an increase of run speed with age. In agreement with previous results also our data shows the highest speed for larvae of age 4 days AEL (Godoy-Herrera et al., 1984). At latest stages the run speed seems to be even reduced (Godoy-Herrera et al., 1984; Figure 2C). For better comparison between the different age groups, we calculated the ratio of run speed to body length (Figure 2D). Surprisingly larvae of the wandering stage have the lowest value for the run speed to body size ratio (Figure 2D). In contrast to the decreased forward locomotion parameters, wandering larvae show an increased pause rate (Figure 2E). The turn rate of wandering larvae seems to be unaffected (Figure 2F). Since larvae during wandering stage seek for an appropriate spot to pupate, we wondered if the reduced speed and the increased pause rate correlated with a change in their locomotion ability. Indeed, a great subset of 6 days AEL larvae showed a drastically decreased forward locomotion and increased pausing (Figures 2G,H). The two example tracks are the composition of all runs and turns a larva with unchanged locomotion (green track, Figure 2G′) and a larva with decreased locomotion (red track, Figure 2G″) were performing respectively during the experiment (Figure 2G). When comparing 5 days AEL and 6 days AEL groups we found that 23% of 6 days AEL animals did not move more than 2.5 cm in any direction away from their starting point, while at 5 days AEL all, except of one, animals moved more than 2.5 cm away from their starting point (Figure 2H). Our results suggest, that larvae of the wandering stage reduce their forward locomotion pre-pupation, whereas turning behavior seems to last normal longer. Moreover, even larvae with extremely reduced forward locomotion show a dark preference. Throughout all tested developmental stages *Drosophila melanogaster* larvae are strongly photophobic.

**Figure 3:**
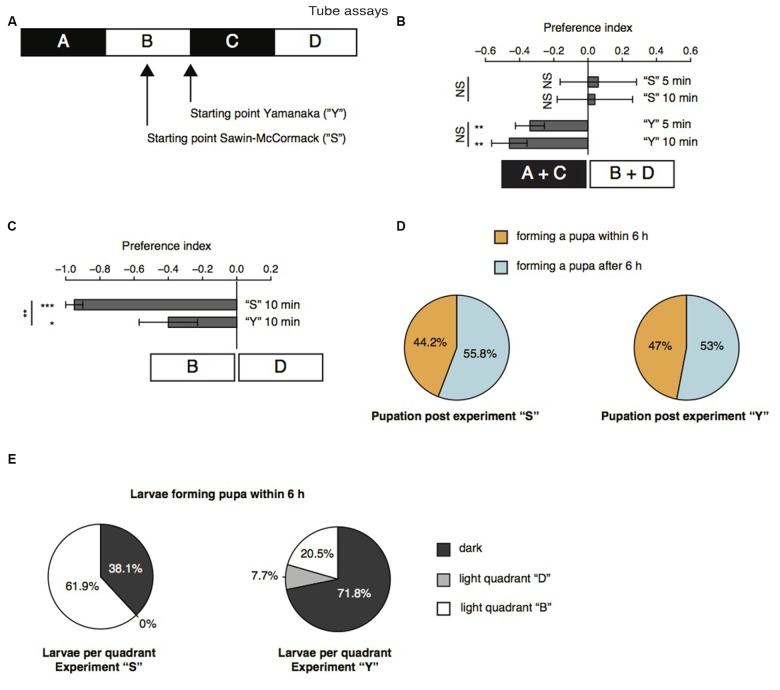
**Age-dependent decreased forward locomotion can impact on the outcome of light preference tests. (A)** Schematic illustration of the tube-assays. Ten larvae were either placed in the middle of section “B” or close to the light-dark boundary. Larvae were stimulated with white light emitting diodes (LEDs) from top and were allowed to move freely in the tubes for 10 min. **(B)** Preference indices of wild-type (WT) 6 days AEL larvae after 5 and 10 min. **(C)** Preference indices of WT larvae for light section “D” against “B”. **(D)** Proportion of larvae forming a pupa within and after 6 h post experiment. **(E)** Proportion of larvae ended up in the different sections. Only larvae were taken into account, which were forming a pupa within 6 h post experiment. Data show the mean and error bars indicate ± SEM. Rejection of the null hypothesis that the mean of the data set is chance or that means of two groups are the same: *p < 0.05, **p < 0.01, ***p < 0.001, One sample *t* test for tests against chance, paired *t* test for testing the preference index after 5 and after 10 min against each other and unpaired *t* test for testing among different groups.

Further, we repeated the previously described tube assays with a few modifications (Sawin-McCormack et al., 1995; Yamanaka et al., 2013). Two apposed glass tubes were fixed together (Figure 3A). We subdivided the glass tubes in four sections by using black tape. The tube-assay possess two dark and two light areas each of them 5 cm in length. The two dark areas were named “A” and “C”, whereas the two light areas were named “B” and “D”. In one experimental sub-set larvae were starting in the middle of section “B” comparable to the experiments described by Sawin-McCormack et al. (1995). In the other experimental sub-set, five larvae were starting in section “B” and five larvae were starting in section “C”, but both as close as possible to the light-dark boundary (Figure 3A). Beside the starting point of the experiments, also the duration of experiments varies between the two tube-assays described earlier (Sawin-McCormack et al., 1995; Yamanaka et al., 2013). Therefore, we counted larvae per section and calculated a preference index after 5 min and after 10 min (Figure 3B). The preference indices after 5 min and after 10 min did not differ from each other in between each behavioral setup (Figure 3B). Thus, the differences in experimental duration of the two tube-assays seems to have no influence on the results. However, different preference indices were calculated depending on the starting point of each experiment (Figure 3B). Experiments in which larvae were starting in the light quadrant led to preference indices of 0.06 and 0.04 for experimental duration of 5 and 10 min respectively. These preference indices are statistically not different from chance and could be interpreted as photoneutral behavior of the larvae (Figure 3B). In case larvae started close to the light-dark boundary, the calculated preference indices were −0.34 and −0.46 for 5 and 10 min experimental duration, respectively. These preference indices were different from chance and suggestive for photophobic behavior of the larvae (Figure 3B). Even though our results of the two experimental set-ups seem to be contradicting to each other, the results are in line with previous reports (Sawin-McCormack et al., 1995; Yamanaka et al., 2013). Larvae starting in the middle of one light quadrant could maybe not been able to leave this quadrant with respect to their decreased forward locomotion (Figure 2). Therefore, we compared the number of larvae found after 10 min in the both light quadrants with each other (Figure 3C). Interestingly, in case the larvae started in light quadrant “B”, we found only one larva in the light quadrant “D”, whereas all the other larvae were found in the starting light quadrant “B” or in darkness. This bias was not as prominent for experiments, in which larvae were starting at the light-dark boundary. However, in both conditions more larvae ended the experiment in section “B” than in area “D” (Figure 3C). These results are suggestive for a great impact of decreased forward locomotion on the tube assays outcome. The decreased forward locomotion could be linked to a pre-pupational state of the respective larvae. Therefore, we calculated how many larvae were forming a pupa within and after 6 h after the end of the experiment respectively. Approximately 45% of the larvae formed pupa within 6 h and around 55% after 6 h post experiment (Figure 3D). These proportions are similar for both assays. Therefore, the differences in preference indices of the two assays cannot be explained by a biased collection of larvae of pre-pupational states for one experimental set. Interestingly, we observed that larvae close before pupation were mainly found in light quadrant “B” in the assay, in which they were also starting in the middle of this light quadrant (Figure 3E). However, in the assay, in which larvae were starting close to the light-dark boundary, larvae of the pre-pupational state were found mostly in the dark area (Figure 3E). Our results suggest, that the reduced forward locomotion, which can be observed in wandering larvae could be linked to a pre-pupational larval state. Moreover, this locomotor phenotype seems to have a great impact on the outcome of the two light preference tube assays.

### Larvae Navigate Robustly Away from Nearly Monochromatic Light Sources with Wavelengths Ranging from UV-A to Green

**Figure 4:**
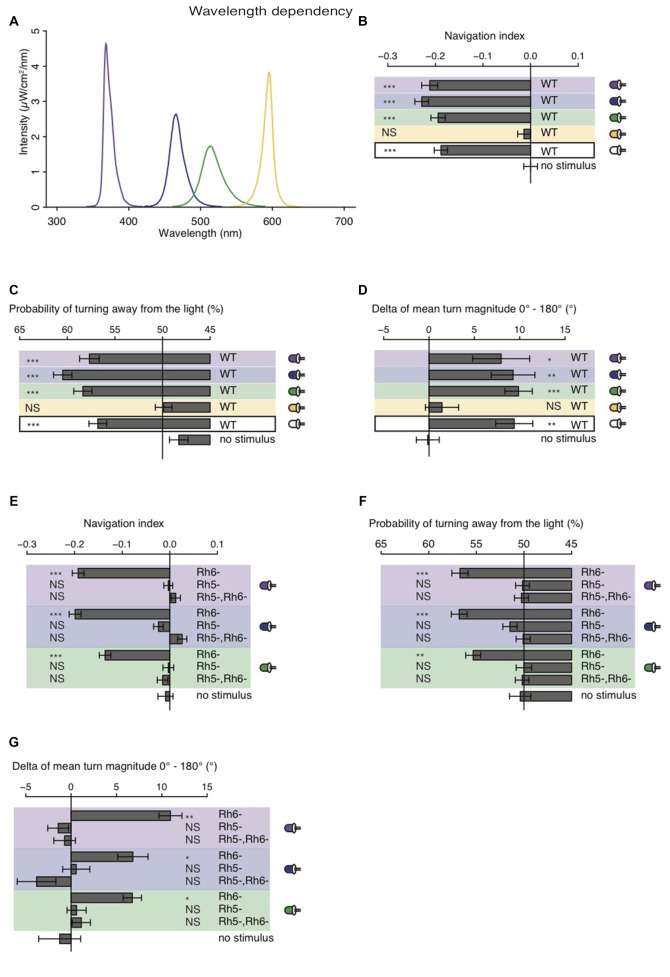
**Light with wavelengths ranging from ultraviolet (UV) to green is robustly avoided by WT and Rhodopsin6 (*Rh6*) mutant larvae. (A)** Spectra of UV (violet line), blue (blue line), green (green line) and yellow (yellow line) light emitted by LEDs, which were used for our behavioral experiments. The different sets of monochromatic and white LEDs emitted light with equal energy levels (69–72 μW/cm^2^). **(B)** Navigation indices of WT larvae stimulated with monochromatic and white light. **(C)** Probability of turn direction of WT larvae after runs heading perpendicular to the different LEDs. **(D)** The delta of reorientation magnitudes of turns where runs were heading previously towards and away from the LEDs. **(E)** Navigation indices of *Rh6* and *Rhodopsin5 (Rh5)* single and double mutant larvae stimulated with monochromatic light. The mutant larvae turn direction probability **(F)** and delta of turn size **(G)**. **(B–G)** Data show the mean and error bars indicate ± SEM. For UV, blue, green, yellow and white light stimulation the data sets are indicated by violet, blue, green, yellow background or black frame respectively. **(B,D,E,G)** Rejection of the null hypothesis that the data set and the no stimulus data set have the same mean: *p < 0.05, **p < 0.01, ***p < 0.001, unpaired *t* test. **(C,F)** Fisher’s exact test was performed to test that the probability of turning away from the light source is significantly different from the turn probability of the no stimulus control: *p < 0.05, **p < 0.01, ***p < 0.001. **(B–G)** The Benjamini Hochberg procedure was performed to adjuste *p*-values.

The larval eye is composed of two PR-subtypes expressing either Rh5 or Rh6, two Rhodopsins with distinct absorption spectra (Salcedo et al., 1999). Rh6 is tuned towards green light with an absorption maximum at 515 nm, while Rh5 is tuned towards blue light with an absorption maximum at 442 nm. The presence of two PR-subtypes with different absorption spectra may thus result in distinct photobehavior, if exposed to different wavelengths of light. Most importantly since previous studies showed that only Rh5-PRs are essential for navigation one might speculate that green light may have no effect on phototaxis, if this green light does not also activate Rh5-PRs. Using a choice assay it was previously described that larvae robustly avoid light with wavelengths ranging from UV-A to green (Warrick et al., 1999). Since the behavioral response to different wavelengths of light of *Rhodopsin* mutants was not studied we here tested WT and *Rhodopsin* mutant larvae using energy-equal (69–72 μW/cm^2^) and nearly monochromatic light emitted by different types of LEDs (intensity peaks at 368 nm, 466 nm, 514 nm and 595 nm; Figure 4A). In consistency with the earlier study we found that WT larvae navigate away from UV-A, blue or green light (Warrick et al., 1999; Figure 4B). In further consistency, yellow light was not avoided by WT larvae (Figure 4B). WT animals were biasing the direction and the size of their turns when stimulated with UV-A, blue, green or white light (Figures 4C,D). WT animals avoid nearly monochromatic light from UV-A to green raising the question how the two PR-subtypes contribute to light-avoidance of different wavelengths. We therefore next tested *Rh5* and *Rh6* single and double mutants. *Rh6* mutant larvae robustly navigated away from nearly monochromatic light ranging from UV-A to green light, whereas *Rh5* mutant larvae and double mutants did not show any navigational response to any light source (Figure 4E). *Rh6* single mutant larvae were able to bias their turn direction and size with respect to the light source (Figures 4F,G). *Rh5* single mutant larvae and the double mutants were not biasing their turn size or direction (Figures 4F,G). Thus, independent of the wavelengths of light Rh5-PRs are necessary for navigating away from light in a light spectrum ranging from UV-A to green light.

### Larvae Navigate Away from the Sun, Even though its Direct Position May be Covered by Clouds

**Figure 5:**
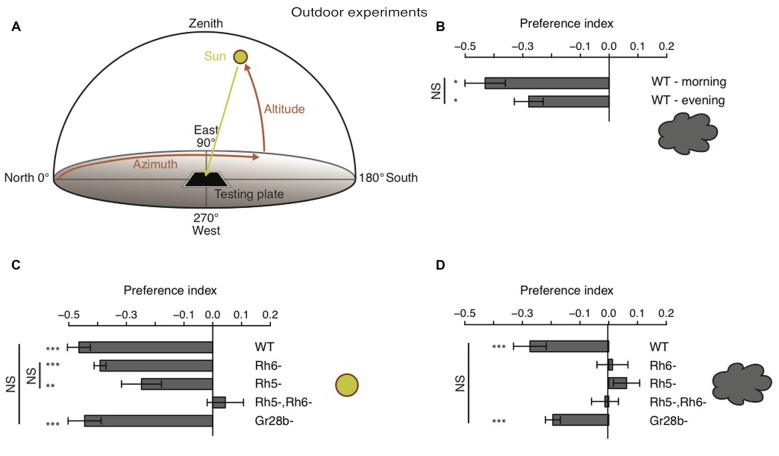
***Drosophila* larvae phototaxis under natural lighting conditions. (A)** Schematic of the outdoor behavioral assay. Azimuth is the horizontal angle from north to the position of the sun. Azimuth defines in which direction the sun is. North has azimuth 0° and east has azimuth 90°. Altitude is the vertical angle from the horizon (0°) to center of the sun and defines the elevation of the sun. Zenith has altitude 90°. **(B)** Preference indices of WT larvae under cloudy conditions in the morning and evening respectivly. **(C,D)** Preference indices of WT, *Rh6* single mutant larvae, *Rh5* single mutant larvae, *Rh5* and *Rh6* double mutant larvae and *Gr28b* mutant larvae stimulated with either direct sun light **(C)** or with diffuse sun light under cloudy conditions **(D)**. Data show the mean and error bars indicate ± SEM. Rejection of the null hypothesis that the mean of the data set is 0: *p < 0.05, **p < 0.01, ***p < 0.001, One sample *t* test. Or rejection of the null hypothesis that the mean of two data set is the same: *p < 0.05, **p < 0.01, ***p < 0.001, unpaired *t* test.

Behavioral studies on phototaxis in *Drosophila* larvae have almost exclusively made use of laboratory assays. Thus, how larvae are able to avoid light in natural lighting condition has not been investigated. It was proposed that larvae could use tropotaxis (spatial integration) to avoid direct (sun) light, while the animals could use klinotaxis (temporal integration) in conditions of diffuse illumination (like on a cloudy day) (Hinnemann et al., 2010). To test if *Drosophila* larvae avoid the sun under both conditions and if also this phototaxis behavior is solely mediated by Rh5-PRs, we performed experiments outdoor either during sunny or cloudy conditions. We therefore used the same testing plate, number of animals and number of repetitions as in experiments under laboratory conditions, however counted the animals in order to calculate a preference index after 20 min (see “Materials and Methods” Section). The sun altitude was not less than 19° and not more than 56° (Figure 5A). Experiments were either performed in the morning when sun azimuth was between 91° and 140° or in the afternoon when the azimuth was between 235° and 261° (Figure 5A). The testing plate was orientated towards the sun position, like this the antisolar side of the testing plate changed position as the sun changes position during a day (Figure 5A). WT larvae were avoiding the solar side on a cloudy day independent of environmental changes due to changes in testing plate orientation (Figure 5B). The preference indices of the morning and evening group did not differ statistically from each other (Figure 5B). Surprisingly, not only WT, but also *Rh6* and *Rh5* single mutant larvae were able to avoid the solar side on sunny days (Figure 5C). Only larvae lacking both *Rhodopsins* did not show a preference for either side (Figure 5C). By testing *Gr28b* mutants, we could further exclude that the avoidance of the solar side is mediated by high light intensity sensing extra-ocular PRs in the larval body wall (Xiang et al., 2010). *Gr28b* mutants behaved like WT larvae (Figures 5C,D). Hence, the Bolwig organ mediates direct sun light avoidance in nature. For the first time, it was shown that not only Rh5- but also Rh6-PRs are sufficient to mediate phototaxis (Figure 5C). In contrast to this result, under cloudy conditions no PR-subtype alone is sufficient for the avoidance of the solar side (Figure 5D). Both PR-subtypes are required for avoiding the solar side, when the sun is covered by clouds (Figure 5D). Under these conditions only the WT control and *Gr28b* mutant larvae were able to avoid the solar side, even though if the preference index was not as strong as under sunny conditions (Figures 5C,D). We therefore conclude, that under natural lighting conditions (direct or diffuse light) both PR-subtypes of the Bolwig organ are jointly required to control negative phototaxis.

## Discussion

### Consistent Phototaxis during Distinct Larval Stages

Adult flies progressively reduce their light preference as they age (Le Bourg and Badia, 1995; Bernardo-Garcia et al., 2016). In the past different hypotheses regarding age-dependent changes or persistence of photobehavior in *Drosophila* larvae were proposed (Manning and Markow, 1981; Markow, 1981; Godoy-Herrera et al., 1992; Sawin-McCormack et al., 1995; Yamanaka et al., 2013). The use of different behavioral paradigms and layouts may offer explanations on differences in behavioral observations. Godoy-Herrera et al. (1992) observed an age-dependent behavioral switch from photophobic to photophilic. They used a two-choice test, in which the dark zone was in the center and the illuminated zone was arranged ring-shaped at the border of the testing plate. Furthermore, the agarose surface was covered with a 2% yeast suspension, in order to prevent the larvae from accumulating at the borders of the plate (Godoy-Herrera et al., 1992). As larvae prefer food in darkness over food in light (von Essen et al., 2011), the observed behavioral switch is likely due to the stop foraging and feeding in late wandering stages and the corresponding search for a pupation site. During this developmental stage, *Drosophila* larvae are leaving the food source, in order to find a suitable pupation site (Bainbridge and Bownes, 1981). This behavior in turn is driven to a great extent by hydrotaxis (seeking for not moisture environment; Rodriguez et al., 1992). Also, Sawin-McCormack et al. (1995) reported that during a comparable light-dark choice assay foraging larvae stayed on the moist agarose, whereas wandering larvae were found on the dry lids of the testing plates, a reason the authors did not use this assay for testing light behavior of wandering larvae. Additionally, larvae appear to be also moderately driven by thigmotaxis (seeking for physical contact), as in quarter or half-plate light-dark preference assays larvae preferably accumulate at the dark edges of the testing plate (data not shown). These two taxes seem to dominate over phototaxis and therefore impact on the results of larval light-dark preference tests.

Sawin-McCormack et al. (1995) also reported, that foraging *Drosophila* larvae avoid light robustly, whereas wandering larvae become photoneutral. This was tested on agarose plates (foraging larvae) or in glass tubes (wandering larvae) with two illuminated and two dark quadrants. At the beginning of the tube-assays experiments wandering larvae were placed into a light quadrant and after 5 min larvae in this and the other light quadrant were counted together (Sawin-McCormack et al., 1995). Each quadrant was spanning over 5 cm of the tube. We found that during terminal wandering stages an increasing portion of animals showed decreased mobility. Thus, a lack of locomotion in the above-mentioned tube-assay may likely cause the observed photoneutrality. Moreover, in a recent study Yamanaka et al. (2013) repeated this tube assay and show that wandering larvae are photonegative and prefer to pupate in darkness. A difference between the two tube assays is, that Yamanaka et al. (2013) let the larvae start at the boundary of light and dark, whereas in the previous study larvae started in the light quadrant (Sawin-McCormack et al., 1995; Yamanaka et al., 2013). We repeated the experiments with the two different tube-assays and our results were in line with both reports. We hypothesize, that larvae with decreased mobility will to a greater probability reach the dark area when starting at the light-dark boundary compared with larvae starting in the light area. At first sight the decreased forward locomotion of wandering larvae resemble sitter behavior in foraging larvae. However, larval rover and sitter phenotypes are food related variations in locomotion of foraging larvae (Sokolowski, 1980; Sokolowski et al., 1997; Allen et al., 2017). The rover and sitter strategies are described as two different approaches of foraging (Sokolowski, 1980). To observe the rover and sitter phenotype foraging larvae, but not wandering larvae, have to crawl on a yeast (food) containing medium. In case no food is present, path length of sitters and rovers are not different from each other (Sokolowski and Hansell, 1992; Sokolowski et al., 1997). The behavioral change in locomotion observed by us is restricted solely to larvae of the wandering stage. These larvae do not forage anymore and do not show rover or sitter behavior. Furthermore, are our experiments performed in absence of food. All the larvae tested in the age-dependency experiments are from the same population. We used nearly all the larvae of each vial and we did not observe reduced locomotion in foraging larvae (excluding a few outliers, 1 out of 300 larvae for 5 days AEL). Taken all this into account, we can exclude rover and sitter behavior as an explanation for the decreased motility in larvae of age 6 days AEL. This age-dependent decreased forward locomotion seems to be due to a pre-pupational stage, because larvae which started and end in the same light quadrant were forming pupa to great extend within 6 h after the experiment. Thus, taking into consideration the type of assay used and the changes in general locomotion and motility during late wandering third instar stages our results strongly suggest that *Drosophila* larvae during all larval stages are photophobic.

### Behavioral Responses to Different Light Spectra

Since *Drosophila melanogaster* is a widely-used laboratory animal model, behavioral studies in a natural environment are sparse. We assessed larval phototaxis by using the same behavioral agar-plates outdoors as we use in highly controlled laboratory conditions. Under sunny and cloudy lighting conditions WT larvae avoid robustly the sun. Phototaxis under these natural conditions are mediated by the larval eye since *Rh5, Rh6* double mutants fail to navigate away from the sun in both cloudy and sunny conditions. Since PRs of the larval eye express the green-tuned Rh6 and the blue-tuned Rh5, it may be assumed that *Drosophila* larvae use the two PR-types to react differentially to different wavelengths of light. In accordance with a previous study (Warrick et al., 1999), our data reveal a wide range of wavelengths which is avoided by *Drosophila* larvae phototaxis. Interestingly, solely Rh5-PRs, but not Rh6-PRs are necessary for this phototaxis in laboratory. Thus, Rh5-PRs are able to perceive light with wavelengths ranging from UV-A to green light. The calculated *Rh5* absorption curve is spanning from 350 nm to more than 510 nm (Salcedo et al., 1999). This corresponds exactly the range of nearly monochromatic light, which is Rh5-PRs-dependent avoided by larvae. Moreover, in transgenic adult flies, electroretinograms revealed that Rh5-PRs or Rh6-PRs robustly respond to stimulation with either blue (430 nm) or green (520 nm) light even though Rh5 and Rh6 possess absorption maxima at distinct wavelengths, their computed absorption curves are overlapping to some extend (Salcedo et al., 1999). In agreement with our findings, these suggest that the two PR-types are rather functioning to mediate or modulate different aspects of navigational behavior than to discriminate colors.

### PR-Types May Contribute to Different Facets of Phototaxis

The complex organization of insect compound eye allows the animal to detect various types of visual cues including color vision, motion detection or polarized light vision (Borst, 2009). An array of ommatidia provides spatial information, while the existence of several distinct PR-types allows for the detection of different light spectra. The visual system of *Drosophila* larvae lacks an ommatidal organization or accessory cells and thus likely only allows a comparison of visual cues between the left and right eye as single spatial points in the visual field. The existents of two PR-types with differently tuned Rhodopsin in principle would allow color-differentiation, however our results suggest that at least for phototaxis there is no wavelength-dependency and only Rh5-PRs are essential for this behavior in laboratory conditions. In natural lighting conditions, we observed that under direct sun light either PR-type is sufficient for phototaxis, while under cloudy conditions both PRs are required. Thus, Rh6-PRs are in fact essential for light-avoidance behavior in natural lighting. The contrary results of the laboratory and the outdoor experiments, led us to the hypothesis, that under natural lighting larvae may use additional or different navigational strategies to avoid the sun and that the two PR-types could mediate distinct strategies. It is proposed that the turn size is based on temporal light information processing (Kane et al., 2013). This temporal processing could happen already in the previous run (before the actual turn starts) or during the actual turn or in both. In case a larva is running perpendicular to a light source a turn towards the light source is as big as a turn away from the light source. This phenomenon is not suggestive for temporal processing during a turn, but for information integration during the previous run. Maybe under natural conditions larvae, which are running perpendicular to the sun, are able to bias the size of turns. Maybe they make smaller turns, when turning towards the sun, and they could make bigger turns when turning away from the sun.

Other authors suggested previously that larvae could use in dependence on lighting conditions klinotacticle or tropotacticle mechanisms (Hinnemann et al., 2010). On a clear day with direct sun light, a larva running perpendicular to the sun could detect the position of the sun (to the left or to the right side) by instantaneously comparing the input of the left and the right eye (tropotaxis; Hinnemann et al., 2010). This hypothesis gets supported by electrophysiological recordings, which demonstrated that in *Calliphora* larvae the larval eyes seems to be directional sensitive due to anatomical features. Lateral light stimulation elicits a much stronger activation of the ipsilateral than the contralateral eye (Hinnemann et al., 2010). As the visual systems of *Drosophila* and *Calliphora* share important anatomical features, a directional sensitivity of the *Drosophila* larval eye seems possible (Hinnemann et al., 2010; Kane et al., 2013). Contrary to this, sun light is more diffuse (less directional) on a cloudy day. By performing head-sweeps larvae could sense temporal changes in light intensity in this diffuse illumination without the need of directional sensitivity (klinotaxis) (Hinnemann et al., 2010). This notion is further supported by the finding that WT preference index is higher under sunny compared with cloudy conditions. Our results are in agreement that phototaxis under sunny conditions could benefit from spatial light cues (for example the directionality of light rays). Since it has been previously shown that larvae can use temporal cues in laboratory conditions for navigational decisions (Kane et al., 2013), we here suggest that depending on the sensory environment larvae may use both spatial and temporal cues for proper phototaxis.

## Author Contributions

T-HH and SGS designed the experiments; wrote the manuscript. T-HH performed the experiments and analyzed the data. SGS coordinated the study.

## Funding

This work was funded by the Swiss National Science Foundation (31003A_149499 to SGS) and the European Research Council (ERC-2012-StG 309832-PhotoNaviNet to SGS).

## Conflict of Interest Statement

The authors declare that the research was conducted in the absence of any commercial or financial relationships that could be construed as a potential conflict of interest.
